# Gross hematuria caused by a congenital intrarenal arteriovenous malformation: a case report

**DOI:** 10.1186/1752-1947-5-510

**Published:** 2011-10-08

**Authors:** Gianpaolo Carrafiello, Domenico Laganà, Gaia Peroni, Monica Mangini, Federico Fontana, Davide Mariani, Gabriele Piffaretti, Carlo Fugazzola

**Affiliations:** 1 Department of Radiology, Ospedale di Circolo e Fondazione Macchi, University of Insubria, Varese, Italy; 2 Department of Vascular Surgery, Ospedale di Circolo e Fondazione Macchi, University of Insubria, Varese, Italy

## Abstract

**Introduction:**

We report the case of a woman who presented with gross hematuria and was treated with a percutaneous embolization.

**Case presentation:**

A 48-year-old Caucasian woman presented with gross hematuria, left flank pain, and clot retention. The patient had no history of renal trauma, hypertension, urolithiasis, or recent medical intervention with percutaneous instrumentation. The patient did not report any bleeding disorder and was not taking any medication. Her systolic and diastolic blood pressure values were normal at presentation. The patient had anemia (8 mg/dL) and tachycardia (110 bpm). She underwent color and spectral Doppler sonography, multi-slice computed tomography, and angiography of the kidneys, which showed a renal arteriovenous malformation pole on top of the left kidney.

**Conclusions:**

The feeding artery of the arteriovenous malformation was selectively embolized with a microcatheter introduced using a right transfemoral approach. By using this technique, we stopped the bleeding, preserved renal parenchymal function, and relieved the patient's symptoms. The hemodynamic effects associated with the abnormality were also corrected.

## Introduction

Renal arteriovenous malformations (AVMs) are rare lesions and may be acquired or congenital. Acquired renal AVMs (arteriovenous fistulas [AVFs]) are relatively rare, accounting for 3% to 5% of all renal AVMs [1]. Hematuria is the major and most common symptom; other clinical manifestations, such as hypertension, left ventricular hypertrophy, cardiac failure, and abdominal pain are also usually associated with AVMs [2]. The usual treatment of AVMs is nephrectomy [3,4], but endovascular embolization can now be considered an alternative [5-8]. We present a case of a congenital renal AVM in a woman who presented to our hospital with gross hematuria and was treated with endovascular embolization in an urgent setting.

## Case presentation

A 48-year-old Caucasian woman was admitted to our hospital with left flank pain and gross hematuria with clot retention. The patient did not report any history of renal trauma, hypertension, known urolithiasis, or recent medical intervention in which percutaneous instrumentation was used. The patient denied any bleeding disorder and was not taking any medication. Her physical examination results were normal, and there was no abdominal bruit on auscultation. The patient's blood pressure was normal at 90/60 mmHg, and her heart rate was 110 bpm.

Her biochemical and coagulation parameters were within normal limits. Urine analysis showed no evidence of leukocytosis, but erythrocytes were present. Urinary system ultrasonography revealed no kidney or bladder lithiasis and no parenchymal or collecting system abnormalities of either kidney.

**Figure 1:**
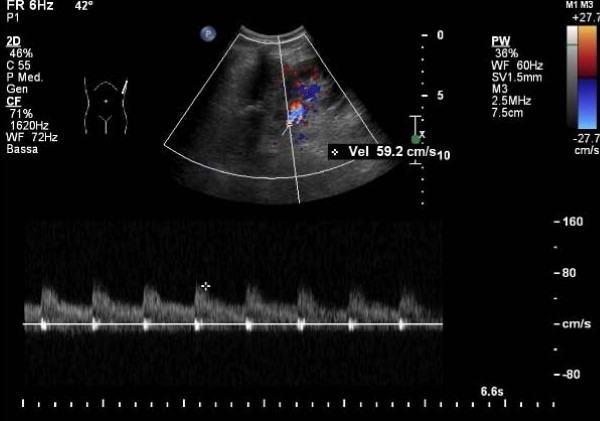
**Spectral Doppler sonogram showing the arteriovenous malformation (AVM)**. The image aliasing area highlights the AVM.

Both computed tomography (CT) and Doppler sonography were performed. Doppler sonography was performed using an IU 22 scanner (Philips, Best, The Netherlands) with a 2 MHz to 4 MHz convex probe. Both color and spectral Doppler sonograms were obtained, which showed turbulent flow with an increased flow velocity of 59.2 cm/second (Figure 1).

**Figure 2:**
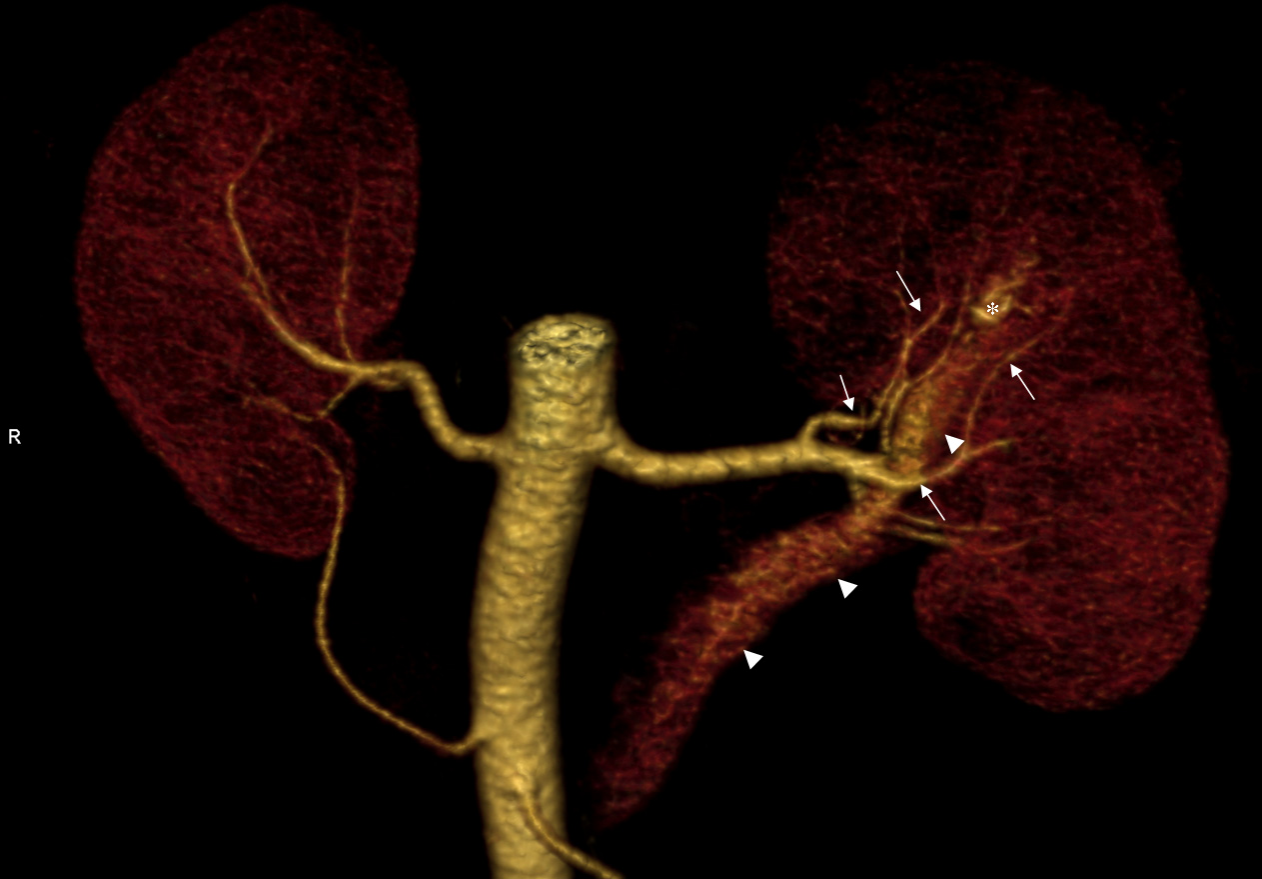
**Computed tomography with iodinated contrast enhancement shows the presence of tortuous blood arterial vessel with thin arterial ramifications of spiral form**. The arrowhead indicates the renal vein, the arrows indicate the renal artery, and the asterisk indicates the renal AVM.

The patient underwent multi-slice CT (MSCT) (Aquilion 64; Toshiba Medical Systems, Tokyo, Japan). After unenhanced CT was performed, 120 mL of iopromide (370 Iomeron; Bracco Imaging SpA, Milan, Italy) was administered using a mechanical injector at a flow rate of 4 mL/second. Biphasic CT was then performed in the arterial phase, and delayed venous phase scanning was performed at a fixed delay of 90 seconds. The CT scan showed the presence of tortuous blood arterial opacified vessels with thin arterial ramifications of spiral form located next to the ileum on the upper pole of the left kidney (Figure 2).

The patient was immediately carried into the angiography room for endovascular treatment. Selective left renal artery angiography was performed using a right transfemoral approach and a 5-French sheath (Terumo Corp., Tokyo, Japan) with a 0.036-inch hydrophilic guidewire coupled with a 5-French cobra-shaped catheter (Cordis, Warren, NJ, USA).

**Figure 3:**
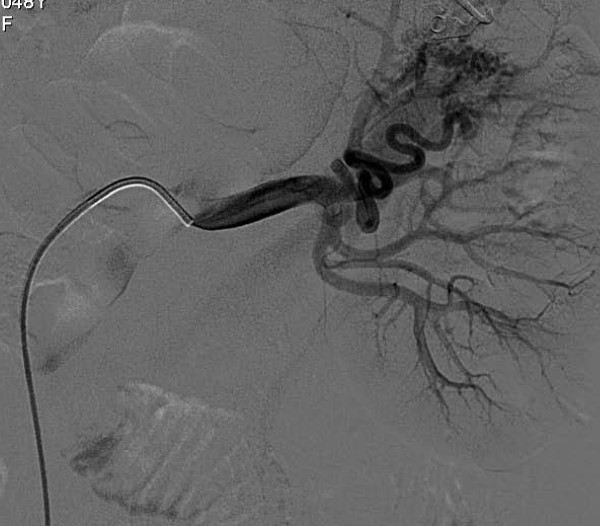
**Selective digital subtraction arteriography of the left kidney showing dynamic images of the AVM**. The black arrow indicates the renal artery, the white arrow indicates the AVM, and the arrows indicate the renal vein.

Digital subtraction arteriography (DSA) demonstrated the feeding artery to the AVM. The lesion was selectively catheterized with a microcatheter (Progreat; Terumo Corp.) and embolized with 4 mm and 3 mm microcoils (Vortex; Boston Scientific, Natick, MA, USA) and microparticles of polyvinyl alcohol 300μ to 500μ and 700μ to 900μ (Bead Block; Terumo Corp.) (Figure 3). No complications occurred during or after the procedure.

**Figure 4:**
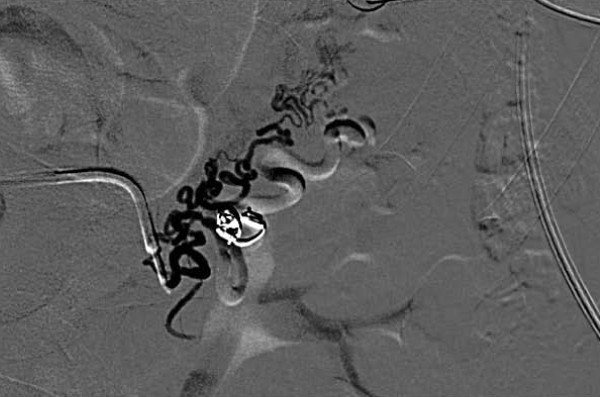
**Digital subtraction arteriography performed after selective embolization of the lesion with the use of microcoils**. Complete excision of the lesion is shown. The renal pelvis is opacified by contrast medium used for arteriography.

At the end of the procedure, complete excision of the AVM was detected using DSA (Figure 4). The patient's hemodynamic parameters, such as blood pressure, were monitored. The patient was discharged seven days later with no signs of hematuria.

## Discussion

AVFs, first described by Varela in 1928 [9], are relatively uncommon lesions with considerable clinical impact. They may cause hypertension, local thrombosis, peripheral embolization, high-output cardiac failure, and hematuria [10].

AVFs can be congenital, acquired, or idiopathic. About 70% to 80% of all AVFs are acquired and may occur as a result of renal biopsy, blunt or penetrating trauma, inflammation, malignancy, or renal surgery [11,12].

AVFs are a congenital condition in 20% to 30% of cases. It is usually located on the kidney upper pole (45%), but it also can be detected in the mid-point or in the kidney lower pole in an equal ratio [13]. The left kidney is more frequently involved, and women are affected twice as often as men. The peak incidence is in patients ages 30 to 40 years, and AVFs are rare in the pediatric population [2].

Acquired fistulas are usually caused by iatrogenic injuries. A fistula can appear after renal needle biopsy, often in kidney transplant patients, and sometimes these fistulas are a post-operative complication after nephrostomy or nephrectomy, particularly in cases of intra-operative injuries of the renal pedicle [14,15].

A fistula caused by angioplasty in a segmental renal artery branch has also been reported in the literature [16]. Malignant tumors of the kidney and metastases can cause fistulas as a result of vein erosion. Other possible causes are penetrating or blunt abdominal trauma, fibromuscular dysplasia, and aneurysm of the renal artery [3,4].

Congenital renal arteriovenous fistulas are the most uncommon form, but their incidence may be underestimated because patients are usually asymptomatic [5,6]. There are two types of congenital AVMs: (1) crisoid, a malformed lesion characterized by multiple varix-like vascular communications and a major incidence of gross hematuria [13], and (2) aneurysmal, which typically occur in elderly patients when a pre-existing arterial aneurysm erodes into an adjacent vein [1].

This kind of malformation has been treated to date with surgical therapy, such as nephrectomy, which is still considered as the first-choice treatment by some authors for patients who present with alterations in the cardiovascular system, such as renin-mediated hypertension caused by fistula-related relative ischemia or high-output cardiac failure caused by an increase in venous return [17]. Endovascular approaches to treating AVMs are now increasingly performed [13].

In our patient, typical diagnostic criteria of the disease were met. The patient was immediately referred to the Department of Radiology for imaging assessments because of her age; moreover, she had received only liquid re-infusions, and neither plasma nor solution of succinylated gelatine (Gelofusine Braun Medical, Milan, Italy) had been administered. It is remarkable that our patient had not undergone any surgical intervention before her presentation to our hospital, which is in contrast to what has been presented in the literature [13].

Our aim was to immediately treat the AVM by performing endovascular embolization to stop the bleeding, preserve renal parenchymal function, and eradicate the symptoms and hemodynamic effects associated with the abnormality that we have seen in our patient, who had a reduction in hemoglobin and an increase in heart rate. It is truly important to preserve renal function in patients who have just one functioning kidney or renal insufficiency [13]. Indications for treating an AVM are a progressive increase in the size of the fistula, recurrent or persistent hematuria, and hemodynamic effects associated with the abnormality, especially decompensation, hypertension, and high-output cardiac failure. Recently, endovascular techniques have also been used to treat giant aneurysms with AVFs. For small renal AVFs, macroparticles or methyl cyanoacrylate glue should be used [5-7]; for larger fistulas, however, coils or detachable balloons are preferable. If there is concern regarding systemic and pulmonary emboli, a high-flow AVF should be managed by performing an open resection or ligation [5-7].

The benefits of percutaneous treatment are avoidance of nephrectomy, reduction of peri-operative risk and post-operative morbidity, reduced surgical time and hospital stay, and decreased incidence of renal ischemia [7].

Post-embolization syndrome (PES) may occur sometimes after transcatheter arterial embolization. PES consists of fever, loin pain, nausea, and vomiting, but selective embolization of renal AVMs allows for the preservation of the renal parenchyma and therefore leads to minimal PES [8].

## Conclusions

Congenital AVMs are uncommon and Color Doppler ultrasonography, MSCT, angiography, and DSA are the most important tools for making the diagnosis in an urgent setting. The therapeutic decision must be made by considering the general condition of the patient and his or her symptoms. The only therapy considered in the past was nephrectomy, but embolization by selective catheterization can be considered safe and effective. However, many studies need to be done to confirm the role of embolization.

## Abbreviations

AVM: arteriovenous malformation; CT: computed tomography; DSA: digital subtraction arteriography; MSCT: multi-slice computed tomography.

## Consent

Written informed consent was obtained from the patient for publication of this case report and any accompanying images. A copy of the written consent is available for review by the Editor-in-Chief of this journal.

## Competing interests

The authors declare that they have no competing interests.

## Authors' contributions

GC, DL, and FF carried out the diagnostic studies and performed the percutaneous embolization. DM and GP reviewed the literature. MM and GP wrote the case report. CF checked and edited the manuscript. All authors read and approved the final manuscript.
